# Real-Time Tracking of BODIPY-C12 Long-Chain Fatty Acid in Human Term Placenta Reveals Unique Lipid Dynamics in Cytotrophoblast Cells

**DOI:** 10.1371/journal.pone.0153522

**Published:** 2016-04-28

**Authors:** Kevin Kolahi, Samantha Louey, Oleg Varlamov, Kent Thornburg

**Affiliations:** 1 Department of Biomedical Engineering, Oregon Health and Science University, Portland, Oregon, United States of America; 2 Center for Developmental Health, Oregon Health and Science University, Portland, Oregon, United States of America; 3 Knight Cardiovascular Institute, Oregon Health and Science University, Portland, Oregon, United States of America; 4 Department of Medicine, Oregon Health and Science University, Portland, Oregon, United States of America; 5 Division of Diabetes, Obesity, and Metabolism, Oregon National Primate Research Center, Beaverton, Oregon, United States of America; Chinese Academy of Sciences, CHINA

## Abstract

While the human placenta must provide selected long-chain fatty acids to support the developing fetal brain, little is known about the mechanisms underlying the transport process. We tracked the movement of the fluorescently labeled long-chain fatty acid analogue, BODIPY-C_12_, across the cell layers of living explants of human term placenta. Although all layers took up the fatty acid, rapid esterification of long-chain fatty acids and incorporation into lipid droplets was exclusive to the inner layer cytotrophoblast cells rather than the expected outer syncytiotrophoblast layer. Cytotrophoblast is a progenitor cell layer previously relegated to a repair role. As isolated cytotrophoblasts differentiated into syncytialized cells in culture, they weakened their lipid processing capacity. Syncytializing cells suppress previously active genes that regulate fatty-acid uptake (SLC27A2/FATP2, FABP4, ACSL5) and lipid metabolism (GPAT3, LPCAT3). We speculate that cytotrophoblast performs a previously unrecognized role in regulating placental fatty acid uptake and metabolism.

## Introduction

**Fig 1:**
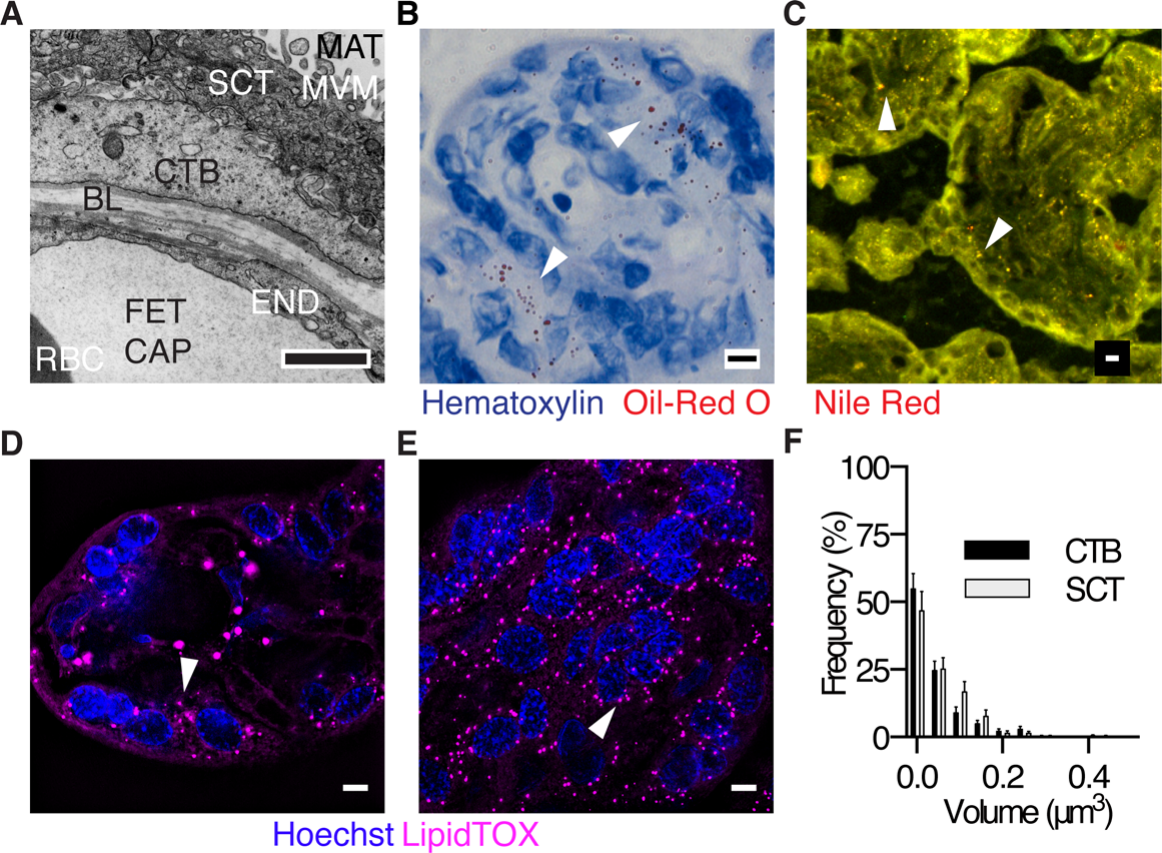
The layers of the human placenta at term and resident lipid droplets. (A) TEM of human placenta at term. MAT, maternal blood space; MVM, microvillous membrane; SCT, syncytiotrophoblast; CTB, cytotrophoblast; END, endothelium; FET CAP, fetal capillary; RBC, red blood cell; BL, basal lamina. Scale Bar: 1μm. (B-E) Lipid droplets (LD, arrow heads) in the human placenta can be detected by multiple staining methodologies. (B) Oil-Red O (C) Nile Red (D-E) Structured illumination microscopy (SIM, a super-resolution technique). Only the syncytiotrophoblast is imaged in (E). (F) The LD volume distribution in freshly delivered placenta is not different (One-way ANOVA) between cytotrophoblast (CTB) and syncytiotrophoblast (SCT) layers, as measured by SIM. (Data are mean ± SEM, n = 7, unpaired t-test). Scale Bar (B-E): 5μm.

The placenta, one of nature’s most important inventions, enables the mammalian fetus to acquire maternal nutrients within the confines of the protective womb. In humans, the placenta is the tissue barrier that separates maternal and fetal bloods and is the gatekeeper for all nutrients acquired by the fetus. The placenta consists of a maternal facing layer of fused cells, the syncytiotrophoblast, an underlying layer of cytotrophoblast cells, a basal lamina, and a fetal capillary endothelium (Fig 1A).

The transport of lipids across the placental barrier is less well understood than the transport of glucose[1]and amino acids[2]. While many known lipid transport proteins are expressed in the human placenta, their roles are little studied. The once held view that all lipids cross the placenta by unregulated diffusion became untenable by reports showing that long-chain polyunsaturated fatty acids (LCPUFA) are transported preferentially[3] and fatty acid transporters exist on the syncytiotrophoblast microvillous membrane[4]. It is now recognized that the fetal acquisition of maternally derived long-chain fatty acids requires a placental transport system[5,6]. Because of our ignorance regarding lipid transport in the placenta and because disruptions in fatty acid supply have dire consequences for fetal brain development[7–10] there is a need for intense investigation into placental lipid transport mechanisms.

Long-chain fatty acid transport begins with the uptake of circulating maternal non-esterified fatty acids liberated from triglycerides in circulating lipoproteins through syncytiotrophoblast lipase activity[11–13]. The fatty acids are translocated into the syncytiotrophoblast by transport proteins (FATP) and fatty acid translocase (FAT/CD36)[4] and shuttled toward the syncytiotrophoblast basal membrane by fatty acid specific binding proteins (FABP) or incorporated within this layer into intracellular compartments.

A large proportion of long-chain fatty acids taken up by the placenta are transformed into esterified glycerolipids (including phospholipids and triglycerides)[14–16]. This is assumed to occur within the syncytiotrophoblast because it contains lipid droplets (LD) and several fatty acid transport proteins in that layer are coupled to an acyl-CoA synthetase enzyme or have acyl-CoA synthetase activity[6,17–19]. The association between long-chain fatty acid uptake and acyl-CoA synthesis has led to the suggestion that the transport of long-chain fatty acids into the fetus includes an intermediate fatty acid esterification step within the syncytium[20,21]. In perfusion studies, the maternal-fetal transfer rates of long-chain fatty acids is directly proportional to degree of placental long-chain fatty acid accumulation[3]. Reduced enrichment of LCPUFA was found in cord blood and placental tissue from gestational diabetic pregnancy *in vivo*[22]. Lower LCPUFA enrichment in cord blood could be due to reduced placental LCPUFA accumulation and esterification, or enhanced fetal LCPUFA uptake [22]. Nevertheless, it remains unclear whether esterification within the placenta serves as a necessary processing step for the regulation of fatty acid transport to the fetus. While the relevance of esterification to fatty acid transport and uptake is debated, it is clear that FATP expression is necessary and proportional to the degree of long-chain fatty acid cellular uptake [23–25].

Before the availability of fluorescent tags and high resolution confocal microscopy, tracking the movement of fatty acids in placental tissue was difficult. Fatty acid uptake and transport studies relied on radiolabeled lipid[26–28] or immunohistochemical methods which cannot localize in real time. In contrast, new confocal visualization techniques reveal that long-chain fatty acids are esterified and sequestered into lipid droplets within hepatocytes [29,30]. These lipid droplets are the principal storage depots for neutral lipids (e.g. triglycerides and cholesterol-esters) in cells[31]. Functionally, LD provides for rapid storage, as well as mobilization, of fatty acids[32]. However, such studies have not been reported in placenta.

We hypothesized that the uptake, esterification and storage of a fluorescent long-chain fatty acid analogue, BODIPY-C_12_, occur within the syncytiotrophoblast layer of the human placenta. BODIPY-C_12_ has an overall chain-length approximately equivalent to that of an 18-carbon fatty acid, and has been used to understand the mechanisms of long-chain fatty acid uptake and trafficking in multiple models[29,30,32–34]. We used real-time optical methods to quantify for the first time, the dynamic processing of BODIPY-C_12_ within the major cell layers of freshly acquired human term placental explants and in isolated cytotrophoblast before and after syncytialization in culture.

## Materials and Methods

All studies were approved by the Oregon Health & Science University Institutional Review Board (IRB# 5684). Written, informed consent for tissue collection was obtained prior to cesarean section surgery.

### Subject Details

Women (>37 weeks gestation, Table 1) scheduled for cesarean section were recruited at Oregon Health & Science University (OHSU) Labor & Delivery. Exclusion criteria included multiple gestations, fetuses with chromosomal or structural anomalies including cardiac defects, preeclampsia, maternal hypertension, and any other significant co-morbidity. Maternal data obtained from medical records included age, parity, race, gestational age at delivery, height, and weight (1st trimester). Neonatal data included birth weight, crown-heel length, and sex. Placenta weight and length and width dimensions were measured during tissue collection.

**Table 1:** Maternal Characteristics.

	Mean±S.D. (n = 30)	95% Confidence Interval
**Age (yr)**	31±6	[29, 33]
**Body Mass Index (BMI: kg/m^2^)**	27±6	[24, 29]
**Parity**	2±1	[2, 3]
**Gestational Age (weeks)**	39±1	[38, 39]
**Birth Weight (g)**	3500±500	[3300, 3700]
**Placenta Weight (g)**	570±140	[500, 630]

### Tissue overview

Thirty placentas were used for different aspects of the study. In brief, placentas were collected at the time of delivery, maternal decidua was removed, and placenta were processed within 30 min. Placental tissues were used as (i) explants for live imaging/trafficking studies, (ii) fixed tissue immunofluorescence studies, (iii) dissociated cytotrophoblast cell studies for *in vitro* live imaging/trafficking studies, or (iv) molecular analysis of mRNA expression. A subset of fresh placental explants were embedded and frozen in OCT cryoprotectant (Cryotek) for immunohistochemistry and LD analyses.

### Placental explant collection

Explants (<1mm3) were isolated as previously described[28], with some modifications. Placental tissue was isolated from two different cotyledons that appeared healthy and placed in M199-HEPES culture media (Gibco) at room temperature (<30mins). Two explants from different cotyledons were cultured per well in plastic 8-well chamber slides (Lab-Tek II, Nunc) containing 0.4mL M199-HEPES and incubated at 37°C in 5% CO_2_/95% air. All explants were assayed within 2-3hrs of delivery, a known window when markers of explant health and fatty acid uptake are not compromised[28,35].

### Primary villous cytotrophoblast isolation and culture

Cytotrophoblast cells were isolated using trypsin-DNAse I digestion followed by Percoll enrichment as previously described[36,37]. In brief, chorion and maternal decidua were removed, and approximately ~50g of villous placenta tissue was finely minced. Villous fragments were subjected to three sequential 30 minute (37°C) digestions in 0.25% Trypsin (Gibco) and 200 U/mL DNAse I (Roche). Cytotrophoblasts were purified by Percoll (GE Healthcare Bio-sciences AB) discontinuous density gradient centrifugation at 1200 rcf for 25 minutes (room temperature). Purity of trophoblast isolations was assessed by positive immunohistochemical staining of cyokeratin-7 (MAI-06315, Thermo Scientific), a marker of trophoblast cells. Contamination by stromal cells and syncytial fragments was determined by staining for Vimentin (PA5-27231, Thermo Scientific) and BHCG (ab9582, Abcam). Our isolations comprised at least >90% pure, viable cytotrophoblasts.

Cytotrophoblasts were plated at a density of 3.5 × 10^5^ cells cm^-2^ on 35 mm imaging dishes (Ibidi USA) compatible with high resolution live-microscopy. Cells were cultured in Iscoves Modified Dulbecco’s Medium (IMDM, Gibco) supplemented with 10% FBS (Gibco) and 100U/ml penicillin and 100μg/ml streptomycin and incubated in a 5% CO_2_/95% air incubator at 37°C. Cell viability was assessed by Trypan blue exclusion. After four hours in culture, non-adherent cells were removed by washing with pre-warmed culture media, and the media was subsequently replaced every 24 hours[37]. A subset of cells was used for live imaging studies, the remainder for qPCR studies. Cells were studied at 4 hours (cytotrophoblasts) or 72 hours (maximum) when most cytotrophoblasts have fused and differentiated into multinucleated, syncytial giant cells[36,38].

### Fluorescent fatty acid tracking and live microscopy (explants, cells)

The use of fluorescently labeled fatty acids to study uptake and LD formation has been adapted from adipocytes[29,30]and applied to placental explants and cells. BODIPY-C_12_ is a long-chain fatty acid analogue that is well established as a tool to study fatty acid uptake and esterification in multiple models[33,34] but has not been previously used in the human placenta. BODIPY-C_12_ is a 12-carbon chain length saturated fatty acid linked to the fluorophore BODIPY (4,4-difluoro-3a,4a- diaza-s-indacene) but the BODIPY-C_12_ conjugate biologically resembles an 18-carbon fatty acid. The BODIPY fluorophore is an intensely fluorescent, intrinsically lipophilic molecule, unlike most other long-wavelength dyes. Chemists argue that probes incorporating this fluorophore are more likely to mimic the properties of natural lipids. BODIPY fatty acids analogues undergo native-like transport and metabolism in cells making it effective as a tracer for lipid trafficking[33,34].

We prepared 10μM solutions of BODIPY-FL C_12_ (BODIPY-C_12_) (Molecular Probes) by diluting a 2.5mM methanol stock solution (1:250) in M199-HEPES containing 0.1% defatted bovine serum albumin (BSA) (Fisher Scientific); solutions were incubated at 37°C for 30mins, protected from light, to allow for BSA conjugation. For most experiments, explants and cells were exposed to BODIPY-C_12_ for 30mins. We sought to determine if exogenously applied fluorescent lipids would be rapidly incorporated into LD within a 30 minute period in human term placenta. The use of the word “rapid” here is not to indicate that placental tissues are able to esterify fatty acids more rapidly than other cells, but to suggest that there may be esterification processes which take much longer than the 30 minute window we studied.

#### Explants

2μL ethidium homodimer (LIVE/DEAD Viability/Cytotoxicity Kit; Molecular Probes) and 1μL of HCS LipidTOX Deep Red (Molecular Probes) was mixed into each well containing placental explants. After 30mins, 100μL of 10μM BODIPY-C_12_ solution was added to each well and mixed by trituration (total well volume 500μL, final concentration 2μM BODIPY-C_12_). To immobilize explants (live or fixed) for confocal microscopy, explants were held to the bottom of the imaging chamber using 8×8 mm pieces of stainless steel mesh (40 Mesh, 0.25 mm; TWP, Inc). For live microscopy, the chamber was also placed into a stage top 37°C incubation chamber. Imaging was performed using a Nikon A1R+/Eclipse TiE resonant scanning confocal microscope equipped with a 60× High N.A. objective (N.A. = 1.4; Nikon). All images were acquired using the same acquisition configurations with minimum laser intensity (0.5%) and exposure time (900ms). Images were acquired every 20 seconds for a total of 30 minutes; there is non-appreciable photobleaching under these conditions. The pixel spacing for all acquisitions was constant and chosen to reflect the Nyquist sampling frequency at 488nm for the magnification objective used. To control for the effect of tissue light scattering, all images were gathered at a consistent distance range (5–10μm) from the culture well base.

#### Cultured cells

Live cytotrophoblast imaging proceeded as above, with a few modifications. Cytotrophoblasts were pre-incubated with Nile Red (1μg/ml; Molecular Probes) for 30 minutes as a counterstain for neutral lipids, and the culture medium replaced with low-fluorescence Dulbecco’s Modified Eagle Medium (Fluorobrite DMEM; Gibco) supplemented with 5% FBS. 2μM BODIPY-C_12_ was added to each well and cells were imaged using a Nikon Eclipse TiE/Yokogawa CSU-W1 spinning disc confocal using a 100× objective (N.A. = 1.49) with 37°C and 5%CO_2_/95% air environmental control. We favored Nile Red for *in vitro* LD labeling because its excitation properties permitted simultaneous excitation of BODIPY-C_12_ and Nile Red with a single laser line, resulting in less photobleaching. We confirmed no detectable bleed-through of neither BODIPY-C_12_ and Nile Red in either channel during the time window studied; the results were similar for LipidTOX. LipidTOX was used in addition to Nile Red as a second dye to ensure interpretation of neutral lipid localization. Nuclei were identified by Hoechst 33342 (0.5μg/ml; Molecular Probes) labeling; multinucleated cells were categorized as syncytiotrophoblast. Cell viability was assessed by Trypan blue exclusion.

### Quantification of total BODIPY-C_12_ uptake in cells

Total BODIPY-C_12_ uptake was determined in cytotrophoblast cultures using a plate-reader assay as described previously[24]. Cytotrophoblast were plated at 3.5 × 10^5^ cells cm^-2^ in 96-well plates (BD Falcon) and cultured as described above. Cells were pre-incubated in 80μL serum free Iscove's Modified Dulbecco's Medium (IMDM) for 60 mins (± inhibitors). After 60 minutes, 20μL of assay solution (25 μM BODIPY-C_12_, 25 μM defatted-BSA, 10mg/ml Trypan Blue) was added to each well, mixed, and incubated for 15 minutes at 37°C with 5% CO_2_/95% air. Fluorescence (485nm excitation, 525nm emission) was measured using a BioTek Synergy H1 hybrid plate reader (BioTek). After fluorescence measurement, the assay medium was removed and cells were carefully washed with Hank’s Buffer for 10min at 37°C. The cells were then lysed using RIPA buffer (EMD Millipore) to measure protein using a BCA method (Pierce).

### Inhibition of fatty uptake and esterification (explants, cells)

BODIPY-C_12_ uptake and fatty acid esterification are dependent on fatty acid transporters (FATP) and long-chain acyl-CoA synthetases (ACSL), respectively. Using a pulse-chase model, explants were exposed to 2μM BODIPY-C_12_ for 2 minutes, then washed with M199-HEPES, and incubated for another 30 minutes before fixation and immunolabeling. Immunolabeling allowed us to localize the fate of the BODIPY-C_12_ pulse after 30 minutes. To test if uptake and fatty acid esterification was sensitive to inhibition of FATPs or ACSLs, explants were co-incubated with 200μM phloretin (nonspecific transporter inhibitor[39]; Sigma) during BODIPY-C_12_ uptake and imaging studies, or preincubated for 1hr with 10μM (in DMSO) triacsin C (long-chain acyl-CoA synthetase inhibitor; Santa Cruz Biotech) or 1hr with specified concentrations of CB-2 (FATP2 inhibitor; EMD Millipore)[24]. The concentrations and incubation times employed are known to block fatty acid uptake and esterification in term explants and cytotrophoblast[40–42].

In explants, we inhibited both syncytiotrophoblast and cytotrophoblast fatty acid transporters and ACSL together; thus we could not conclude whether reductions in LD production were due to inhibition of uptake or esterification in the syncytium, or solely in the cytotrophoblast. To test if the production of BODIPY-C_12_ LD in cytotrophoblast was independent from the syncytiotrophoblast we performed complementary *in vitro* experiments in primary isolated term villous cytotrophoblasts. Cells were studied at 4 hours (cytotrophoblasts) and also at 72 hours after the cells had spontaneously fused and differentiated to form syncytial giant cells[38]. Co-localization was assessed in cultured cells at 15 minutes during continuous 2μM BODIPY-C_12_ incubation with or without phloretin or triacsin C. We used live spinning disc confocal microscopy together with Nile Red to track LD[30] to determine whether alterations in cytotrophoblast LD production are independent of changes of uptake or esterification in the syncytium.

### Immunofluorescence (whole mount explants, cells)

Explants were fixed in 4% paraformaldehyde (pH 7.4) for 20 minutes at room temperature before washing 3 times in phosphate buffered saline (PBS, pH 7.4), and stored in PBS at 4°C for up to 48 hours. Samples were blocked and permeabilized in 5% normal goat serum (Life Technologies)/0.1% saponin (Sigma) for 1 hour before overnight incubation with primary antibody at 4°C. Samples were labeled with antibodies against BHCG (1:100; ab9582, Abcam), E-cadherin (1:200; ab11512, Abcam), HAI-1 (1:100; 9B10, eBioscience), FATP2 (1:50; 14048-1-AP, Proteintech), Perilipin-2 (1:100; ab52355, Abcam), and Perilipin-3 (1:100, PA5-20272, Thermo Scientific). Following primary antibody incubation, samples were washed 3 times with 0.1% saponin in PBS and labeled with secondary antibodies (donkey F(ab’)2 anti-mouse Alexa Fluor 647 (1:1000, ab150103), donkey F(ab’)2 anti-rat Alexa Fluor 555 (1:1000, ab150150), donkey F(ab’)2 anti-rabbit Alexa Fluor 568 (1:500, ab175694), donkey F(ab’)2 anti-rabbit Alexa Fluor 647 (1:1000, ab150103), and donkey anti-rabbit Alexa Fluor 488 (1:1000, ab150069); all secondary antibodies from Abcam) for 1 hour at room temperature. After washing 3 times, the samples were counterstained with 1μg/ml Hoechst 33342 for 10 minutes and immersed in Slowfade Diamond (Molecular Probes) immediately before confocal imaging. Slowfade Diamond did not disrupt LD morphology and photobleaching resistance was substantially improved over samples imaged in PBS. Imaging was performed using a Nikon A1R+/Eclipse TiE resonant scanning confocal microscope equipped with a 60× High N.A. objective (N.A. = 1.4; Nikon).

### Structured Illumination Microscopy (SIM) Imaging (explants)

Our initial estimates by confocal fluorescence microscopy of LD diameter (~600nm) were near the diffraction resolution limit for light (Airy disc diameter = 560nm; wavelength = 640nm, N.A. = 1.4). Thus, measurements by standard optical techniques may overestimate the true size of an LD. Hence we used structured illumination microscopy (SIM) which affords, under ideal conditions, a 2-fold increase in the lateral spatial resolution and is compatible with conventional fluorophores such as LipidTOX. Using SIM, our theoretical Airy disc diameter is 280nm (wavelength = 640nm, N.A. = 1.4). Since the volume of a spherical LD scales with the cube of its radius, a small increase in lateral resolution can afford a dramatic increase in the minimum detectable volume of LDs; SIM under ideal conditions permits more accurate measurement of particles 8-fold smaller than possible using conventional confocal fluorescence microscopy.

For super-resolution microscopy of LD via SIM, explants were stained with LipidTOX Deep Red. Structured illumination microscopy was performed using an Elyra PS.1 (Zeiss) microscope equipped with a 63× objective (N.A. = 1.4). Thin (0.1 μm) z-stacks of 20 high resolution images were collected in five rotations and reconstructed using Zen software (Zeiss). All processed images were analyzed using Fiji[43].

### Image Analysis

All images obtained via confocal and SIM imaging were analyzed using Fiji software[43]. LD were automatically segmented using the Renyi Entropy method, and quantified using consistent particle analysis parameters in Fiji and are expressed as a number (abundance) normalized to the acquired 3-D image volume of syncytiotrophoblast or cytotrophoblast. In all immunofluorescence explant experiments, replicates represent at least 2 explants, each with at least 2 independent fields of view. For live imaging of explants, 2 separate explants were imaged with one field of view that demonstrated exclusion of ethidium homodimer dead stain. On average about 90 LD were measured per replicate using SIM imaging. In live-cell imaging experiments, each time point represents the mean of a maximum-intensity projection of 20 z-slices (0.2 μm thick) representing a 3-D volume with 4μm total thickness. With the plating density utilized, about 10 nuclei were present in a single 3-D volume on average. Co-localization analyses were performed using Imaris (Bitplane AG) and Fiji using the automatic thresholding method of Costes et al.[44] to obtain a Pearson co-localization coefficient; this coefficient indicates the degree to which BODIPY-C_12_ is being incorporated into the LD organelles identified by Nile Red or LipidTOX[30].

### qPCR gene expression

A subset of primary cytotrophoblasts were used for gene expression studies; these cells were not used for imaging studies. Total RNA from 3 × 10^6^ cells was isolated after 4hr or 72hr of culture using Qiagen RNeasy isolation kit. RNA content was assessed by spectroscopy at 260nm/280nm and integrity via visualization of ribosomal RNA using gel-electrophoresis. Reverse transcription of 750ng of RNA to cDNA was performed using the High Capacity cDNA Reverse Transcription kit (Life Technologies). cDNA was stored at -20°C. Gene specific primers were designed for fatty acid transporter 2 (*SLC27A2*), and long-chain acyl-CoA synthetase 1 (*ACSL1*), -3 (*ACSL3*), and -5 (*ACSL5*), fatty acid binding proteins 4 (*FABP4*), -5 (*FABP5*), 1-acylglycerol-3-phosphate O-acyltransferase 9 (*GPAT3*), lysophosphatidylcholine acyltransferase 3 (*LPCAT3*), and BHCG (*CGB*) using NCBI primer-BLAST[45]. Sequences for primers for GAPDH, fatty acid transporters 1 (*SLC27A1*), -4 (*SLC27A4*) and fatty acid translocase (*CD36*) were obtained from Brass et al. and Mishima et al.[28,46]. Primer sequences and gene information are shown in Table 2; qPCR was performed as described previously[47]. PCR amplicons were detected by fluorescence of Power SYBR Green (Applied Biosystems) and following manufacturers recommendations using the Stratagene Mx3005P Thermocycler (Agilent Technologies). Cycling conditions were the same for all primer pairs with 42 amplification cycles. For each primer pair, a standard curve, no template controls and unknowns were run in triplicate. Following cycling, the melt curve of the resulting amplicon was analyzed to ensure that a single product was detected for every replicate. Efficiency of each primer set was calculated using the slope of the respective standard curves with manufacturer’s software (MxPro v4.10; Stratagene) and the predicted product size for each primer pair was verified via gel-electrophoresis. The cycle threshold (Ct) was calculated for each replicate using MxPro software for detecting the amplification-based threshold. No replicates were excluded and all replicates were within 0.5 SD of the average Ct. Relative expression was quantified using comparative quantification[48]. The same sample was used as the “calibrator” in all assays. Relative expression quantities were expressed as a ratio of the gene of interest to the reference gene (*GAPDH*) in each sample; *GAPDH* expression did not differ at 4 hours versus 72 hours.

**Table 2:** Primer sequences utilized for quantitative PCR.

Gene	Function	Accession	Forward (5’→3’)	Reverse (5’→3’)
ACSL1	Long-chain fatty acid acyl-CoA synthetase	NM_001995	GGGCAGGGAGTGGGCT	TCTAAGCTGAATTCTCCTCCGTG
ACSL3	Long-chain fatty acid acyl-CoA synthetase	NM_004457	GGCGTAGCGGTTTTGACAC	CCAGTCCTTCCCAACAACG
ACSL5	Long-chain fatty acid acyl-CoA synthetase	NM_016234	CTGAAGCCACCCTGTCTCTG	AGGAAATTCAGACCCTGCGA
GAPDH	Reference gene	NM_002046	AGGTGGTCTCCTCTGACTTC	TACTCCTTGGAGGCCATGTG
GPAT3	*De novo* glycerolipid synthesis	NM_001256421	GAGGGCCTCCAGGTGAGT	AGAGACACTCCGAAGACCGA
CD36	Fatty acid transporter	NM_001001548	AAACCTCCTTGGCCTGATAG	GAATTGGCCACCCAGAAACC
BHCG (CGB)	Marker for cytotrophoblast syncytialization	NM_00737	ACCCCAGCATCCTATCACCT	CACGCGGGTCATGGTG
CPT1A	Fatty acid transporter located on mitochondrial outer membrane	NM_001876	ATTACGTGAGCGACTGGTGG	TGTGCTGGATGGTGTCTGTC
FABP4	Fatty acid binding protein	NM_001442	AAACTGGTGGTGGAATGCGT	GCGAACTTCAGTCCAGGTCA
FABP5	Fatty acid binding protein	NM_001444	TGAAGGAGCTAGGAGTGGGAA	TCTGCCATCAGCTGTGGTTT
LPCAT3	Glycerophospholipid synthesis	NM_005768	TTCCTGGGTTACCCCTTTGC	GCCGGTGGCAGTGTAATAGT
SLC27A1	Fatty acid transporter	NM_198580	CCGGAATTGACTGTGACCACTT	CACGCAGTGCAGGGTTCA
SLC27A2	Fatty acid transporter	NM_003645	TGCTGCACTACTGATTGGCA	TTGGTTTCTGTGGTGAGTTGC
SLC27A4	Fatty acid transporter	NM_005094	GCTGCCCTGGTGTACTATGG	GGAGGCTGAAGAACTTCTTCC

### Electron Microscopy

Electron microscopy of term placental tissue was performed as previously described[49]. Placental pieces were fixed in 2.5% glutaraldehyde/2% formalin in 0.1 M phosphate buffer for 45 minutes. The tissue was then sliced into thinner pieces and allowed to fix for an additional 30 minutes. Lastly, the tissue was cut to embedding size and fixed for 30 minutes before being placed in 0.1 M phosphate buffer, pH 7.3, 4°C overnight. The next day, the tissue blocks were post fixed for 90min in 2% osmium tetroxide, dehydrated in ethanol series and embedded in Epon resin. Electron micrographs were made on JEOL electron microscopes.

### Lipid Extraction and Thin Layer Chromatography

The incorporation of BODIPY-C_12_ into lipid classes was characterized as described previously [33]. Cells were incubated with 5μM BODIPY-C_12_ in 6-well dishes and were collected and washed with HBSS containing 0.1% BSA at specified time points. Lipids were extracted using chloroform and the lipid phase was collected and evaporated using a vacuum concentrator (SpeedVac). Lipid residues were dissolved in 100μL of chloroform and applied onto silica gel 60G TLC plates (EMD Millipore). Neutral lipids were separated on TLC plates using heptane/isopropyl ether/acetic acid, 60:40:4, vol/vol/vol). Developed plates were visualized under UV light (365nm) using an emission filter (515-570nm) and images were captured using a digital camera.

### Statistics

ANOVA with Bonferroni post-hoc testing was used to test for differences in LD abundance via SIM in LD analyses (one-way) and BODIPY-C_12_ LD accumulation in each cell type (2-way). Total BODIPY-C_12_ uptake and qPCR were analyzed by a paired t-test. A four parameter, nonlinear regression analysis was used to calculate IC_50_ values for CB-2 FATP2 inhibitor. All data were analyzed using GraphPad Prism 6 and are presented as mean ± SEM unless noted otherwise. P-values <0.05 were considered statistically significant.

### Study Approval

Studies were approved by the Institutional Review Board (IRB # 5684). Written, informed consent for tissue collection was obtained prior to cesarean section surgery.

## Results

### Lipid droplet (LD) analysis in explants

The existence and localization of LDs in human term placenta was confirmed in all 30 placentas using several modalities of microscopy and multiple staining methods (Fig 1B–1E). LDs were visible in the syncytiotrophoblast, cytotrophoblast and fetal endothelium. LD volumes in cytotrophoblast (0.047±0.006 μm^3^) were similar to syncytiotrophoblast (0.038 ± 0.003 μm^3^) (Fig 1F, N.S.). Likewise, LD abundance was not different between cytotrophoblast (3×10^7^ ± 4×10^6^ LD mm^-3^) and syncytiotrophoblast (3×10^7^ ± 5×10^6^ LD mm^-3^). We found no associations between LD volume or abundance with fetal sex in syncytiotrophoblast or cytotrophoblast. These data suggest that LD are a normal feature of all human term placentas. The presence of LD in normal placenta, led us to speculate that the syncytiotrophoblast was capable of free fatty acid esterification and that LD in cytotrophoblast originated from those manufactured in the syncytium. We also supposed that the transfer of LD from syncytial tissue to deeper layers in the placenta could be visualized in real-time using microscopic methods.

#### BODIPY-C_12_ uptake in live and fixed explants

**Fig 2:**
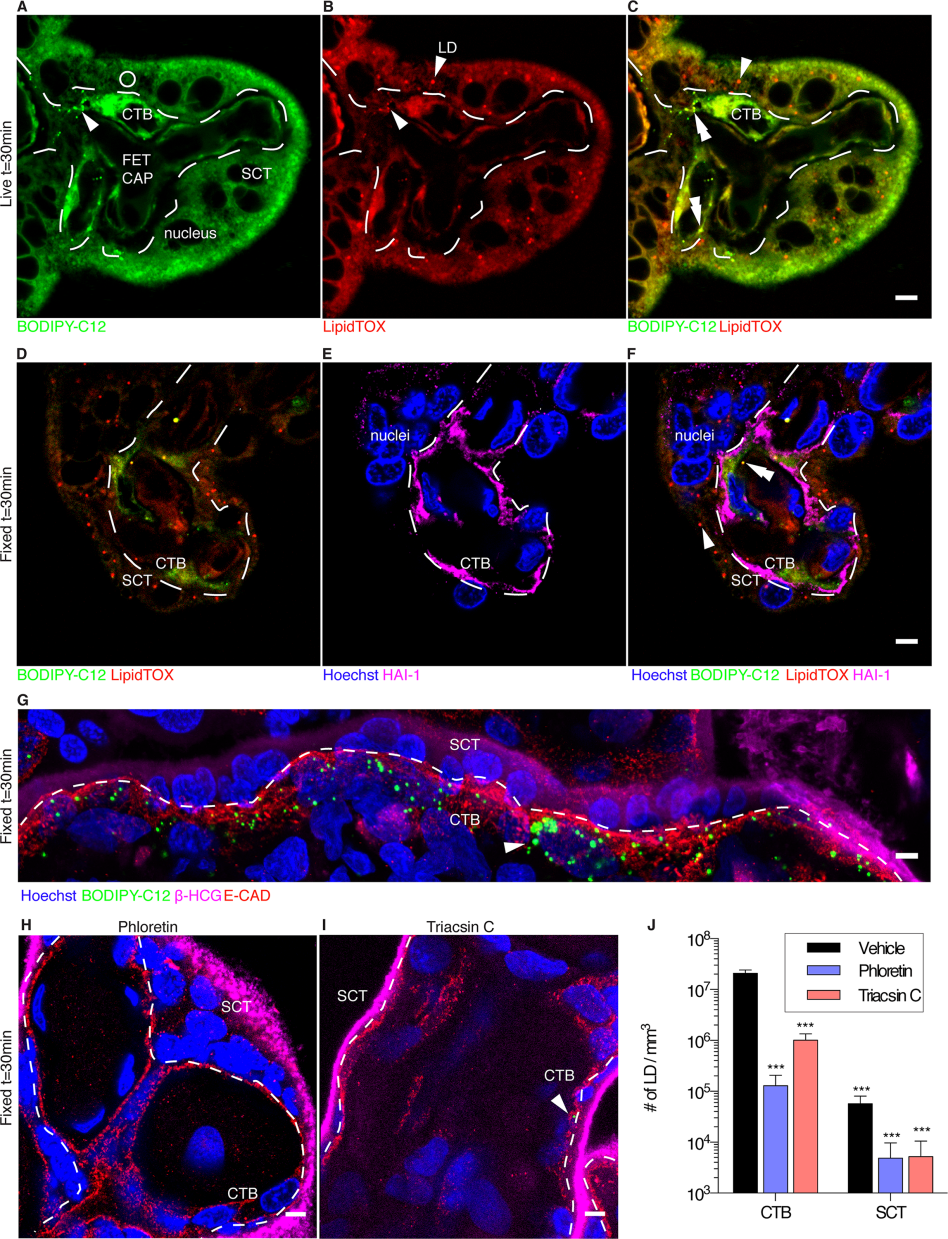
BODIPY-C_12_ fatty acid uptake in human term placental explants (30 min exposure). Dashed line represents the syncytiotrophoblast-cytotrophoblast interface. Co-localized BODIPY-C_12_ and LipidTOX forms yellow LD, indicating esterified BODIPY-C_12._ (A) BODIPY-C_12_ (green) is localized to punctate structures (arrow heads) and diffuse areas in syncytiotrophoblasts (SCT) and cytotrophoblasts (CTB). Dark “holes” are unstained nuclei. (B) Pre-existing neutral lipids (LipidTOX, red) are found in punctate structures which are lipid droplets (LD) (arrow head); (C) LipidTOX co-localizes with BODIPY-C_12_ (double arrowhead) predominantly in CTB, providing evidence that BODIPY-C_12_ is esterified and localized within LD. Conversely, in SCT, LD (arrowhead) contains little BODIPY-C12 (corresponding region 2A open circle). (D-F) images of explants after fixation showing BODIPY-C_12_ (green) and LipidTOX (red). (D) LD are present in both SCT and CTB, but BODIPY-C_12_ seen in (A) has mostly washed out in processing but some remain in sequestered organelles. (E) Hoechst (blue) marks nuclei and HAI-1 (magenta) marks CTB, (F) BODIPY-C_12_ (green) is found primarily in CTB (magenta) but not in SCT (see double arrow head). (G) BODIPY-C_12_ containing lipid droplets (arrow heads) are found extensively in the CTB layer (e-cadherin, red) but are sparse in the SCT layer. (H) phloretin (a blocker of protein mediated transport), and (I) triacsin C (a blocker of long-chain fatty acyl-CoA synthetases) reduces the population of lipid droplet containing BODIPY-C_12_ in CTB. These experiments suggest that transporters and acyl-CoA synthetases are required for production of LD. (J) Quantification of lipid droplets synthesized in explants illustrating the effects of chemical inhibitors and shows under control conditions BODIPY-C_12_ LD accumulation is higher in 30 min in the CTB vs. SCT; (I): Data are mean ± SEM; n = 6 control, n = 5 phloretin, and n = 4 triacsin C. 2-way ANOVA, *** = p<0.001 vs CTB vehicle; VS, villous stroma; FET CAP, fetal capillary; Scale Bar: 5μm.

We sought to determine whether exogenous fluorescent-labeled BODIPY-C_12_, a long-chain fatty acid analogue, would be incorporated into syncytiotrophoblast LD of living explants within 30 minutes, a technique and time period that has been used in other tissues including white adipose tissue[29]. Uptake and distinct localization patterns of BODIPY-C_12_became evident within 30 min. BODIPY-C_12_ appeared both diffusely and as punctate structures within the cytoplasm of placental tissue, which we surmised to be the syncytium and the cytotrophoblast (Fig 2A).

LDs are composed of endogenous neutral lipids (triglycerides and cholesterol-esters)[31] that can be visualized by LipidTOX staining (Fig 2B). As indicated by merged staining, BODIPY-C_12_ was incorporated into preexisting LipidTOX positive LD within 20 minutes, signifying that (Fig 2C) it was being esterified, a requirement for LD incorporation[33]. Surprisingly, esterification was consistently associated with the cell layer beneath the syncytiotrophoblast, which appeared to be cytotrophoblast (Fig 2C and 2D; S1 Movie, S2 Movie). To test this, placental tissues were stained with hepatocyte growth factor inhibitor type 1 (HAI-1), a cytotrophoblast-specific marker [50]. It confirmed that the cells containing newly esterified BODIPY-C12 were cytotrophoblasts (Fig 2E). The cytotrophoblast layer appeared to be rarely interrupted under our microscopic conditions and was the layer that rapidly accumulated esterified BODIPY-C_12_ (Fig 2G). In separate experiments, explants fixed after 30 min also confirmed co-localization of BODIPY-C_12_ with LipidTOX in LD organelles within the cytotrophoblast, but not within the syncytiotrophoblast (Fig 2D–2F), in which there was little co-localization between the markers even by 60 minutes (not shown).

In a separate set of experiments, placental tissues were exposed to a 2 minute pulse of BODIPY-C_12_, followed by washout, to determine the fate of fatty acids initially absorbed by the syncytiotrophoblast. From previous experiments, we determined that incorporation of BODIP-C_12_ into LD required 30 minutes. We measured LD production after initial uptake using this time point. Thirty minutes after the 2 minute pulse, the abundance of BODIPY-C_12_ labeled LD was higher in cytotrophoblast than syncytiotrophoblast (2×10^7^ ± 3×10^6^ LD mm^-3^ versus 6×10^4^ ± 2×10^4^ LD mm^-3^; p<0.001, Fig 2J). The number of BODIPY-C_12_ LD in cytotrophoblast was dramatically reduced following treatment with the nonspecific transporter inhibitor, phloretin, (1×10^5^ ± 8×10^4^ LD mm^-3^; p<0.001, Fig 2H and 2J) and the long-chain acyl-CoA synthetase inhibitor, triacsin C (1×10^6^ ± 3×10^5^ LD mm^-3^; p<0.05, Fig 2I and 2J), implying both FATP and ACSL are involved in the rapid production of BODIPY-C_12_ LD. In contrast, LD abundance in the syncytiotrophoblast was not altered by phloretin (5×10^3^ ± 4×10^3^ LD mm^-3^) or triacsin C (5×10^3^ ± 5×10^3^ LD mm^-3^, Fig 2J).

#### BODIPY-C_12_ uptake in live, isolated undifferentiated cytotrophoblast

**Fig 3:**
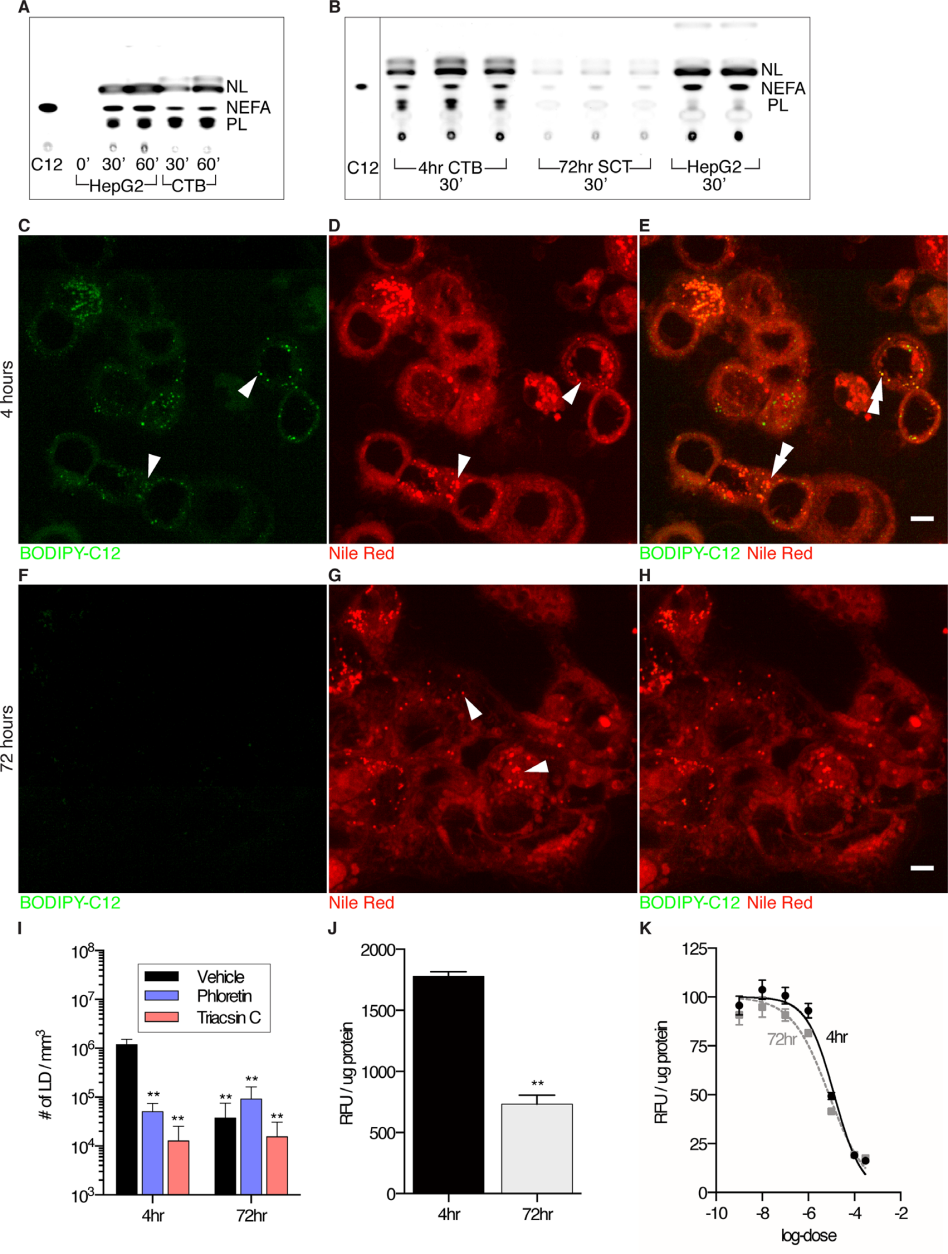
BODIPY-C_12_ incorporation into lipid droplets in primary cytotrophoblasts incubated for 15 or 30 min. Cytotrophoblasts (CTB) were in two states of maturation after being in culture for 4hr(A,C-E) or 72hr which leads to syncytialization (SCT) (B,F-H). (A,B) Analyses of BODIPY- C_12_ incorporation into lipid subclasses via thin-layer chromatography in CTB and SCT *in vitro* (A) Time course of HepG2 cells (positive control) and primary cytotrophoblast incubated continuously with BODIPY-C_12_. BODIPY-C_12_ is incorporated into neutral lipids (NL) and phospholipids (PL) within 30 minutes indicating esterification of this long-chain fatty acid analogue. NEFA, non-esterified fatty acid. (B) CTB incorporate more BODIPY-C_12_ into esterified lipid subclasses than SCT *in vitro* within 30 minutes. (D) Cultures (D,E and G,H) were stained with Nile Red to visualize neutral lipid droplets (LD). Images (C,F) shows that BODIPY-C_12_ incorporation is greater among CTB than among SCT. (E) In CTB, BODIPY-C_12_ merges with Nile-Red in LD. (H) When CTB have undergone syncytialization, they incorporate very little BODIPY-C_12_ in LD. (I) Quantification of lipid droplets shows that cultured (4 hours) cells accumulate more BODIPY-C_12_ LD than those that have syncytialized (72hrs). Phloretin and triacsin C significantly reduce BODIPY-C_12_ LD accumulation in cytotrophoblasts but not in syncytialized cells. (J) The uptake of BODIPY-C_12_ using a 96-well plate-reader indicates that uptake is higher in cytotrophoblast before syncytialization (K) The log-dose response curve of CB-2, a FATP2 inhibitor, supports that both cytotrophoblast and syncytialized cells are equally sensitive to inhibition of BODIPY-C12 uptake by CB-2. The efficacy of this inhibition of BODIPY-C_12_ uptake suggests that FATP2 largely mediates long-chain fatty acid uptake in these cells. TLC plates are representative of 3 technical replicates. (C-H): Scale Bar: 5μm, LD (arrow heads), co-localization (double arrowhead). Image intensity ranges are the same in all panels (I-K): Data are mean ± SEM; (I) n = 6 for each group. 2-way ANOVA, (J,K) n = 4. Paired t-test. * = p<0.05, ** = p<0.01 vs CTB vehicle.

Cytotrophoblasts are unique in that they are isolated in their immature undifferentiated form but become mature over time in culture to form multinucleated syncytialized giant cells, presumably as *in vivo*. We used the differentiation process to determine whether the capacity to esterify fatty acids was lost during differentiation and syncytialization. To test if esterification was taking place within cytotrophoblast, we analyzed BODIPY- C_12_ incorporation into lipid subclasses via thin-layer chromatography in isolated primary cytotrophoblast. We found that BODIPY-labeled neutral lipids were present within 30 minutes (Fig 3A), and that differentiation of cytotrophoblast (4hr) to syncytiotrophoblast (72hr) led to a pronounced decrease in esterified BODIPY-C_12_ esterification products (Fig 3B). This finding is consistent with the observation that BODIPY-C_12_ is esterified during integration into cytotrophoblastic LDs and that differentiation results in a switch in lipid metabolic phenotype.

We measured total uptake of BODIPY-C_12_, using a plate reader before and after cytotrophoblast differentiation/syncytialization. BODIPY-C_12_ Uptake was approximately 2-fold greater in cytotrophoblast at 4hr (1.8×10^3^ ± 37 RFU /μg protein) than in syncytial giant cells at 72hr (7×10^2^ ± 74 RFU /μg protein; p<0.01, Fig 3J). We also tested the degree to which BODIPY- C_12_ uptake in cultured trophoblast was sensitive to CB-2, an inhibitor of FATP2. FATP2 is the most highly expressed fatty acid transporter in the placenta[42]. The similar efficacies and estimates of IC_50_s of CB-2 indicates 4hr cytotrophoblast and 72hr syncytial giant cells are not differentially sensitive to CB-2 (4hr, 13 ± 2.4 μM) vs (72hr, 8 ± 1.6 μM; Fig 3K) and thus both of these cell types are likely dependent on FATP2 for the uptake of BODIPY-C_12_ long-chain fatty acid.

Primary villous trophoblast accumulates BODIPY-C_12_ LD at different rates depending on the degree of maturation. Cytotrophoblasts (4hrs, Fig 3C–3E) had greater abundance of BODIPY-C_12_ LD than syncytialized cells (72hrs, Fig 3F–3H) (1×10^6^ ± 0.3×10^6^ LD mm^-3^ vs 4×10^4^ ± 1×10^5^ LD mm^-3^; p<0.01, Fig 3I). Consistent with our explant experiments, BODIPY-C_12_ LD abundance was reduced by phloretin (5×10^4^ ± 6×10^4^ LD mm^-3^; p<0.01) and triacsin C (1×10^4^ ± 3×10^4^ LD mm^-3^; p<0.01) in cytotrophoblasts (Fig 3I) and with minimal effect on syncytial giant cells (Fig 3G).

**Fig 4:**
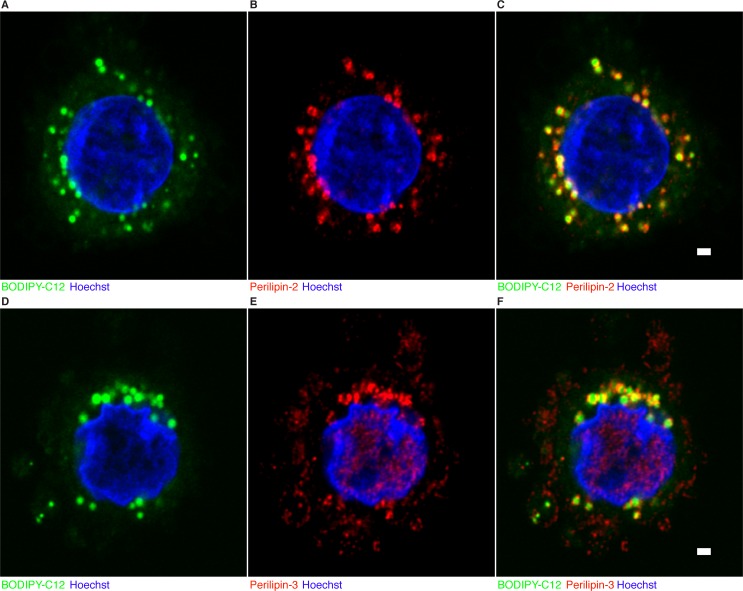
BODIPY-C_12_ incorporates into Perilipin-2 and Perilipin-3 labeled cytotrophoblast lipid droplets. Cytotrophoblast plated for 4hr onto glass coverslips were incubated with 2μM BODIPY-C_12_ for 30 minutes and fixed as described in the methods section. Cells were immunolabeled with Perilipin-2 or Perilipin-3. Nuclei are labeled using Hoechst dye. All BODIPY-C_12_ cytotrophoblast lipid droplets contain some degree of Perilipin-2 (A-C) or Perilipin-3 (D-F) on the surface. In addition to the presence of Perilipin-3 on lipid droplets, Perilipin-3 is also distributed on the plasma membrane of cytotrophoblast (E). n = 5. Scale Bar: 1μm.

To test if the BODIPY-C_12_ LD are labeled by LD-associated proteins, we fixed and immunolabeled 4hr CTB after 30 minutes of incubation with BODIPY-C_12_. We found all BODIPY-C_12_ LD in CTB contained some degree of LD associated Perilipin-2 or Perilipin-3 (Fig 4). We detected little BODIPY-C_12_ LD synthesis in 72hr syncytialized cells.

#### Lipid transporter gene expression in cultured cytotrophoblasts

**Fig 5:**
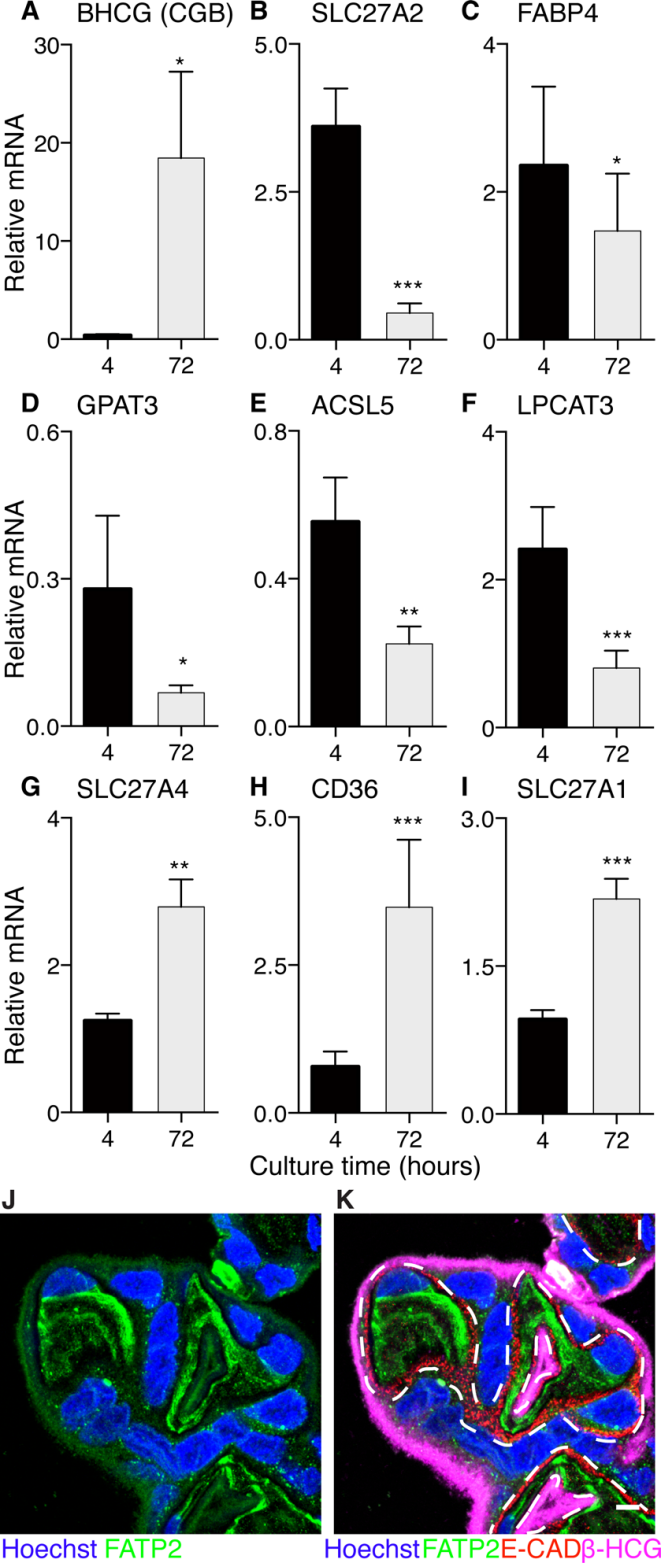
mRNA expression of key lipid processing genes before and after cytotrophoblast syncytialization. (A) In 72hr syncytia, the expression of *BHCG* is significantly higher compared to cytotrophoblast at 4hrs, indicating differentiation. (B-I) By 72 hours, there are significant changes in expression of several fatty acid uptake and processing genes. (B) *SLC27A2* (FATP2, the most highly expressed fatty acid transport protein in placenta). (C) *FABP4* (fatty acid binding protein). (D) *GPAT3* (glycerol-3-phosphate acyltransferase). (E) *ACSL5* (Acyl-CoA synthetase, long-chain). (F) *LPCAT3* (lysophosphatidylcholine acyl transferase). (G) *SLC27A4* (FATP4, fatty acid transport protein). (H) *CD36* (FAT, fatty acid translocase). (I) *SLC27A1* (FATP1, fatty acid transport protein). Data are mean ± SEM, n = 6 for all groups. Expression is relative to GAPDH, which is not significantly different between groups. Paired t-test * = p<0.05, ** = p<0.01, *** = p<0.001. (J) Immunofluorescence of FATP2 (green), nuclei are blue (Hoechst). (K) Merged immunofluorescence illustrating cytotrophoblast (E-cadherin, red), syncytiotrophoblast (BHCG, magenta) with FATP2/SLC27A2 (green). FATP2 can be found in both cytotrophoblast and syncytiotrophoblast, but appears to be more strongly expressed in cytotrophoblast which fits gene expression data in panel (B). Dashed line represents the syncytiotrophoblast-cytotrophoblast interface. Scale Bar: 5μm.

After 72hrs, *BHCG* mRNA expression in cultured cytotrophoblasts was >4500% higher (p<0.05), indicating differentiation into syncytial giant cells; *BHCG* is a specific marker of syncytialization. We also looked for the presence of desmoplakin, which disappears from intercellular junctions with syncytialization. Junctional complexes were absent in syncytial giant cells as indicated by the loss of desmoplakin S1 Fig. Concomitant with cytotrophoblast syncytialization, there was a pronounced decrease in *SLC27A2* expression, the gene encoding FATP2 (-88%, p<0.001). Genes regulating glycerolipid metabolism were downregulated during syncytialization: *GPAT3* (-75%, p<0.05), *ACSL5* (-60%, p = 0.002), *FABP4* (-38%, p<0.05), and *LPCAT3* (-66%, p<0.001). In contrast, mRNA levels of *SLC27A4* (FATP4; +133%, p<0.1), *CD36/FAT* (+349%, p<0.001), and *SLC27A1* (FATP1; +129%, p<0.01) increased during syncytialization. We did not detect differences in of *FABP5*, *ACSL1*, *ACSL3*, or *CPT1A* expression (data not shown). FATP2 (*SLC27A2*) is highly expressed in the placenta and we found it is distinctly localized to the cytotrophoblast (Fig 5J and 5K) perhaps within a discrete subcellular compartment.

## Discussion

We tested the hypothesis that BODIPY-C_12_ would be incorporated into esterified lipid pools within syncytiotrophoblast. We chose BODIPY-C_12_ as a tracer because it is well-established tool to investigate long-chain fatty acid uptake and esterification in many cell types[33,34]. The strength of the method is that fatty acid uptake can be tracked in real-time within different cell layers and sub-cellular compartments of the placenta.

Contrary to our hypothesis, the syncytiotrophoblast was not the primary site of esterification of this synthetic long-chain fatty acid. Rather, BODIPY-C_12_ esterification occurred almost exclusively in the cytotrophoblast. We were surprised to find that the syncytiotrophoblast seemed to be incapable of esterifying BODIPY-C_12_. We were forced to conclude that the cytotrophoblast plays a previously unsuspected role in placental lipid metabolism.

It has been previously reported that the cytotrophoblast layer, found beneath the syncytial layer becomes increasingly interrupted as gestation proceeds[51]. This view contributed to the conclusion that cytotrophoblast plays an insignificant role in transport processes as term approaches. In contrast, Mori et al. suggested that the cytotrophoblast cell layer is nearly continuous over most of the villous placental surface until term[50]. Furthermore, although the layer gets thinner toward term, the total number of cytotrophoblast cells increases, by approximately 6 fold, between the first and third trimester[52].

Lipid droplets have been biochemically detected in placenta and isolated by others[18]. In our study, we were able to visualize LD in all term human placentas examined using an array of microscopic techniques (Fig 1). Because high concentrations of non-esterified fatty acids can be cytotoxic, incorporation into LD offers safe storage and a way to regulate fatty acid trafficking[53]. We did observe a portion of exogenous BODIPY-C_12_ that was taken up by cytotrophoblast but not incorporated into pre-existing LD within the time frame of our experiments. The fate of the unincorporated fatty acid fraction is not known. In similar experiments performed in hepatocytes, there are pre-existing LDs that immediately incorporate BODIPY-C_12_ akin to our neutral LDs in placenta, but these cells also contain a secondary LD population that arises within minutes after being incubated with BODIPY-C_12_[30]. Like the hepatocyte experiments, we found new lipid droplets in cytotrophoblast within minutes of exposure to BODIPY-C_12._ Our live imaging studies were limited to 30–60 minute windows to ensure that tissues were metabolically stable[30]. Alternatively these structures may represent sites of phospholipids incorporating BODIPY-C_12_, as we have observed BODIPY-C_12_ incorporation into CTB phospholipid pools during esterification (Fig 3).

BODIPY-C_12_ may approximate some features of long-chain fatty acids, but further tests are warranted to delineate how these results translate to the esterification of natural long-chain fatty acids in trophoblast. Primary cultured human cytotrophoblast cells are highly active in esterification and lipolysis of ^14^C radiolabeled oleic acid[21] but show a 2–3 fold decrease in fat esterification and glycerolipid synthesis after 72 hours in culture[21]. Our findings point to a decline in esterification occurring concurrently with syncytialization.

Although LD are present in the syncytium, we did not find evidence that the esterification took place within the syncytiotrophoblast. Thus, we speculate that the syncytiotrophoblast acquires LD manufactured in the cytotrophoblast. The transfer could occur by fusion of cyto- to syncytio-trophoblast under repair conditions. It is well known that cytotrophoblasts are incorporated into areas of syncytium to replace damaged regions of syncytiotrophoblast [51]. In addition, the LD could be transported directly from the cytotrophoblast to the syncytial layer, an attractive but untested explanation for the large numbers of LD in syncytiotrophoblast.

*SLC27A2* (FATP2) is the most highly expressed fatty acid transporter (FATP) in the human term placenta[42]. The uptake and processing of BODIPY-C_12_ correlates more with its expression, than with *SLC27A4* (FATP4) or *ACSL1*[25]. FATP2 is found within multiple subcellular locations including peroxisomal membranes, cytosolic plasma membrane, and endoplasmic reticulum, and serves a variety of roles in lipid metabolism including mediating LCPUFA uptake[23]. In culture, *SLC27A2* had higher levels of mRNA expression and higher staining intensity in cytotrophoblasts at stages than after the cells became syncytialized. However, the expression level of transporters may or may not directly equate to to transporter activity levels, and the role of post-translational FATP modifications in placental fatty acid uptake is unknown[54].

We blocked FATP2 function with CB-2 to determine the degree to which FATP2 was responsible for transporting BODIPY-C_12_ into trophoblast whereupon BODIPY-C_12_ uptake was reduced by ~80%. We determined that IC_50_ values for CB-2 in trophoblast were comparable to those reported for cells expressing high levels of FATP2, Caco-2 and HepG2 (IC_50_~10μM)[24] but were much lower (20-fold) than for 3T3-L1 adipocytes that primarily express FATP1[24]. The potency and efficacy of CB-2 on uptake suggests FATP2 may be largely responsible for driving BODIPY-C_12_ uptake in trophoblast. FATP2 can drive long-chain fatty acid uptake via its acyl-CoA synthetase activity[23]. This process displays selectivity for LCPUFA and is known to channel fatty acids into esterified lipid pools[23]. The low level of FATP2 expression in syncytiotrophoblast suggests that it is not well suited for long-chain fatty acid acylation.

A number of other key fatty acid transporters and glycerolipid metabolic genes were more highly expressed before syncytialization than after. These patterns of expression may be tied to the differing roles of cytotrophoblast and syncytiotrophoblast. Glycerol-phosphate acyl transferase-3, encoded by the *GPAT3* gene, catalyzes the initial and rate limiting step in the *de novo* formation of triglycerides [55]; *ACSL5* is expressed in tissues with high rates of triglyceride synthesis[56]. Thus higher expression of these genes in cytotrophoblast is consistent with greater neutral lipid and LD synthetic capacity. *LPCAT3* preferentially channels LCPUFA, including omega-3 fatty acids, into cellular phospholipids[57]. Higher levels of all of these genes support the observation that there is greater capacity for fatty acid esterification in cytotrophoblast. Alternatively, differential esterification in cytotrophoblast may be accounted for by greater availability of other glycerolipid precursors (e.g. glycerol, acyl-glycerols, and phospholipids) which we did not measure.

Cytotrophoblast LD incorporation of BODIPY-C_12_ could be disrupted by triacsin C and phloretin. Reductions in LD production due to Triacsin C may indicate ACSL(1,3,4, or 5) involvement[58–60], while phloretin sensitivity may indicate a role for protein mediated transporters or a requirement for ATP, since phloretin disrupts multiple cellular transporters including GLUT1 [61].

Our study was not designed to address the relevance of esterification in regulating the transplacental transport of free fatty acids. However, long-chain fatty acids taken up by the placenta are found predominantly incorporated into esterified lipids, and esterification is hypothesized to be an intermediate step during fatty acid transport in the placenta[20,21]. Our study does illustrate differing lipid dynamics between the cytotrophoblast and syncytiotrophoblast and suggests that future considerations of placental fatty acid transport include studies on the role of cytotrophoblast in addition to syncytiotrophoblast. In fact, the LD in the cytotrophoblast may represent the metabolically active lipid pool that was hypothesized to exist in a recent model of human placental fatty acid transfer[62]. Esterified fatty acids in trophoblast can be re-released via lipolysis or exported after incorporation into apolipoprotein particles [63], but the mechanisms by which these processes occur have not been described in placenta. Our BODIPY-C_12_ uptake and FATP-2 expression data are also consistent with the idea that long-chain fatty acids are more highly sequestered in and permeable to cytotrophoblast than the syncytiotrophoblast. Transplacental movement of fatty acids likely involves an interplay between these two cell layers.

Understanding the fundamentals of fatty acid transport and storage in the placenta is highly important because the human fetus must acquire large amounts of fat to provide for its developing brain; inadequate amounts of omega-3 LCPUFAs result in cognitive deficits in offspring [22,64]. The prevalence of obesity, gestational diabetes mellitus and pre-eclampsia is increasing in the US, conditions associated with dysregulation of LCPUFA uptake, esterification and transfer, and altered fusion and viability of cytotrophoblasts[22,65–67]. Understanding the mechanisms that underlie these dysfunctions and developing interventions to prevent them will require a robust understanding of normal fatty acid transport in the placenta.

## Conclusions

**Fig 6:**
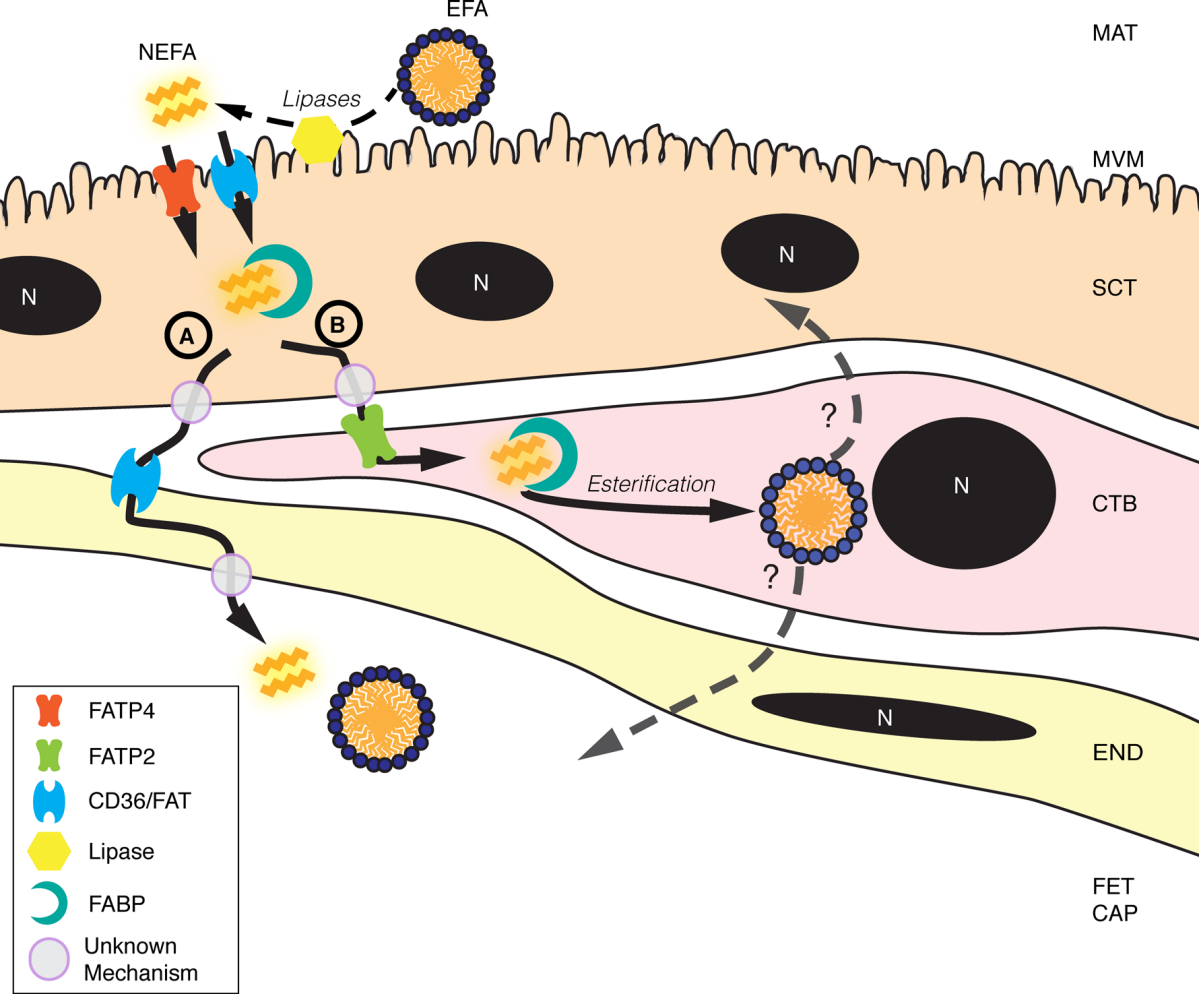
Hypothetical simplified model of placental transport of fatty acids.

Using novel optical methods we have found differential localization of LD synthetic activity in the two trophoblast layers of the term human placenta. Based on these new findings we propose a modified model of lipid uptake and storage in the placenta (Fig 6). The cytotrophoblast had not previously been reported to play a significant role in long-chain fatty acid processing. Differential rates of esterification may due to inherent differences in expression of FATPs and ACSLs in the two cell types. During syncytialization of the cytotrophoblast, multiple genes involved in esterification and glycerolipid processing are significantly downregulated, which supports the observed differences in LD production in cytotrophoblast compared to syncytiotrophoblast.

Long-chain fatty acids are rapidly absorbed into villous tissue after lipolytic activity by lipases on triglycerides at the microvillous membrane. Fatty acid transporters/translocases (FATP, CD36) appear to facilitate the uptake of long-chain fatty acids, but the importance of fatty acid activation and conjugation to Acyl-CoA during transport is not known. Intracellular fatty acids or fatty Acyl-CoAs are held by fatty acid binding proteins (FABP). The mechanism of fatty acid efflux is unclear, but free fatty acids may proceed through may proceed to fetal capillaries (FET CAP) in a manner resembling facilitated diffusion. This route (Route A) does not involve esterification. Unlike previous explanations of long-chain fatty acid transport, our data suggest an additional route B. Non-esterified fatty acids on Route B are transported across the syncytial layer and taken up by cytotrophoblast where they are rapidly esterified and incorporated into lipid droplets. LD in SCT may derive from CTB. The mechanisms by which the long-chain polyunsaturated fats versus saturated fats are differentially transported are unknown. Nevertheless, long-chain fatty acid transport processes are dependent upon a number of lipases and transport proteins, including FATP2, FATP4, FABP, and CD36. MAT, maternal blood space; MVM, microvillous membrane; SCT, syncytiotrophoblast; CTB, cytotrophoblast; END, endothelium; FET CAP, fetal capillary.

## Supporting Information

S1 Fig Syncytialized cytotrophoblast contain few detectable BODIPY-C_12_ LDs. Cytotrophoblast cells cultured for 72hr were incubated with 2uM BODIPY-C_12_ for 30 mins, fixed, and immunolabeled with desmoplakin (1:200, ab16434, Abcam) as described in the methods section. Nuclei are stained with Hoechst dye. Imaging was performed using a Zeiss 880 LSM Confocal with Airyscan. The absence of desmoplakin intercellular divisions (red) indicates that these trophoblast have syncytialized. Very little BODIPY-C_12_ or few LDs are evident in this syncytialized cell. n = 5. Scale Bar: 1μm.
(TIF)

S1 Movie Time lapse confocal movie of human term placental explant over 30 minutes. BODIPY-C_12_ (green) is incorporated into cytotrophoblast lipid droplets (LipidTOX Far Red, red), but very little in syncytiotrophoblast. Time (min:sec). Dashed line represents the syncytiotrophoblast-cytotrophoblast interface. Magnification: 600X, Scale Bar: 5μm.
(AVI)

S2 Movie Time lapse confocal movie of human term placental explant over 30 minutes over a larger field of view. This video illustrates a larger field of view and scans through the villus depth (0.2μm min^-1^). Similar to Movie S1, (BODIPY-C_12_ (green) is incorporated into cytotrophoblast lipid droplets (LD, LipidTOX Far Red, red), but very little in syncytiotrophoblast. Multiple cytotrophoblast are visible sequestering BODIPY-C_12_ into LD in this larger field of view (arrow heads). Time (min:sec). Magnification: 600X, Scale Bar: 5μm.
(AVI)
