# Supplementary figures and images for: Real-Time Tracking of BODIPY-C12 Long-Chain Fatty Acid in Human Term Placenta Reveals Unique Lipid Dynamics in Cytotrophoblast Cells
Source: PLoS One. 2016 Apr 28;11(4):e0153522. doi: 10.1371/journal.pone.0153522 (PMC4849650; doi:10.1371/journal.pone.0153522)

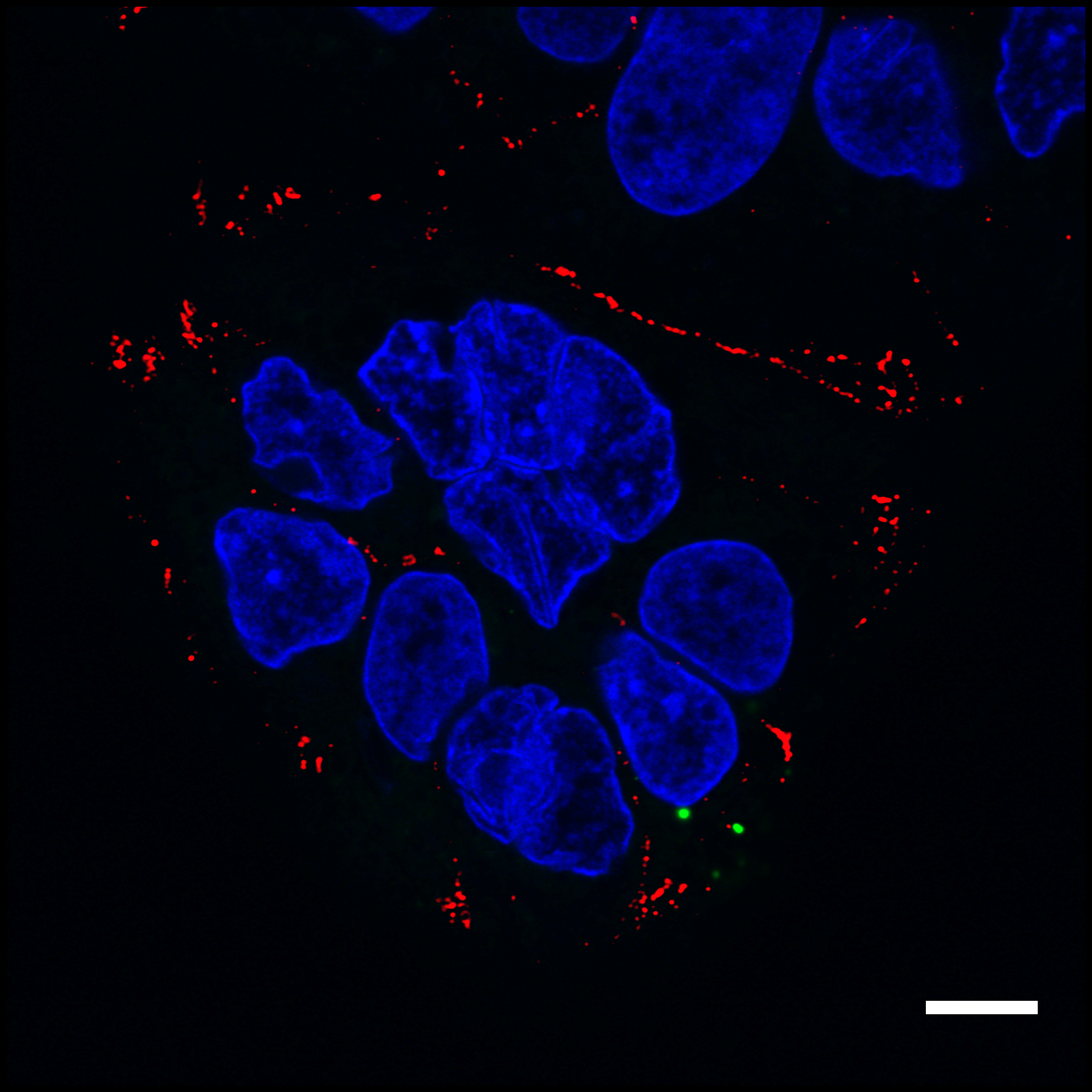

Supplement: S1 Fig — Cytotrophoblast cells cultured for 72hr were incubated with 2uM BODIPY-C12 for 30 mins, fixed, and immunolabeled with desmoplakin (1:200, ab16434, Abcam) as described in the methods section. Nuclei are stained with Hoechst dye. Imaging was performed using a Zeiss 880 LSM Confocal with Airyscan. The absence of desmoplakin intercellular divisions (red) indicates that these trophoblast have syncytialized. Very little BODIPY-C12 or few LDs are evident in this syncytialized cell. n = 5. Scale Bar: 1μm. (TIF) [file pone.0153522.s001.tif]
